# Unexpected Anatomical Variation While Performing an Ultrasound-Guided Interscalene Block for Shoulder Surgery

**DOI:** 10.7759/cureus.25079

**Published:** 2022-05-17

**Authors:** Mohamed Fayed, Suzana Khalil, Nimesh Patel, Adnan Hussain

**Affiliations:** 1 Anesthesiology, Perioperative Medicine, and Pain management, Henry Ford Health System, Detroit, USA

**Keywords:** brachial plexus anatomic variation, scalene muscle, abnormal anatomy, acute pain management, peripheral nerve stimulator, total shoulder arthroplasty, ultrasound anatomy, ultrasound guided regional anesthesia, ultrasound guided nerve block, interscalene nerve blocks

## Abstract

Anatomical variations of the brachial plexus are very common. Knowledge of the possible anatomical variations encountered in ultrasound imaging is crucial for the safe and effective practice of regional anesthesia. The interscalene block (ISB) targets the brachial plexus roots in the interscalene groove, between the anterior and middle scalene muscles (MSM), at the level of the sixth cervical vertebra. Blockade of the brachial plexus roots anesthetizes the shoulder region, making the ISB one of the preferred regional anesthesia options in shoulder surgeries. Abnormalities of the muscular structures surrounding the brachial plexus roots can pose a challenge while performing an ultrasound-guided ISB. We present a case of an unanticipated anatomical variation of the anterior scalene muscle (ASM) encountered on ultrasound imaging when performing an ISB. Our patient was found to have a small redundant ASM, which necessitated an alternative scanning approach and the use of a nerve stimulator to properly identify the brachial plexus roots. Based on our findings, we recommend placing the ultrasound probe parallel to the clavicle in the supraclavicular area and scanning in a cranial direction, tracing the brachial plexus back to the roots, and then confirming the needle placement by using a traditional nerve stimulator before local anesthetic deposition.

## Introduction

An interscalene block (ISB) refers to the deposition of local anesthetics around the brachial plexus roots in the interscalene groove at the level of the sixth cervical vertebra. The carotid artery is identified through an ultrasound-guided technique. The brachial plexus is then found laterally, between the anterior scalene muscle (ASM) and middle scalene muscle (MSM), and deep to the prevertebral fascia, superficial cervical plexus, and sternocleidomastoid muscle. Anesthetizing the brachial plexus at this level provides sensory and motor blockade of the shoulder and upper lateral arm, making the ISB a preferred choice in regional anesthesia for distal clavicle, shoulder, and proximal humerus surgeries. The most common complications from an ISB are diaphragmatic hemiparesis, which occurs in almost 100% of cases due to ipsilateral phrenic nerve blockade, vocal hoarseness from ipsilateral recurrent laryngeal nerve blockade, and Horner's syndrome from ipsilateral stellate ganglion blockade [1]. ISB is contraindicated in patients with severe respiratory disease as previously mentioned, as it has been associated with ipsilateral phrenic nerve paralysis in 100% of cases. Also, caution should be exercised in patients with potentially difficult airways as well, since performing an invasive airways procedure could be challenging for anesthesiologists [2]. There is also evidence to suggest that ISB may transiently increase the regional brain oxygen saturation levels on the ISB side in the sitting position during shoulder surgery [3].

Prior research has frequently described anatomical variations of the brachial plexus. A study of 51 cadavers showed that the classically described anatomy of the brachial plexus situated in the interscalene groove between ASM and MSM was only present in 60% of the cadavers, the most common anomaly being the penetration of the ASM by C5 or C6 nerve roots. It also showed that in 3% of cadavers, the C5 nerve root was passing anterior to the ASM [4]. Another cadaveric study performed on 93 cadavers showed five different anatomical variations: the upper trunk was found perforating the ASM in 6.45% of cases; C5 was passing anterior to the ASM in 3.22% of cases; the upper trunk was running anteriorly to the ASM in 2.15% of cases; the upper trunk was passing between the two bellies in 0.54% of cases; and C5 was passing anteriorly to the ASM while C6 was perforating the ASM in 0.54% of cases [5]. Previous research has also shown that muscular variations exist in muscular bridges between the ASM and the MSM or connections between the superficial and deep muscle layers [6]. This muscle variation may predispose individuals to symptomatic nerve entrapments [7].

Although prior studies and case reports have focused on the anatomical variations of the brachial plexus roots, there is scant data on the anatomical variation of the musculature, its clinical implications, and how it can pose a challenge to performing a safe and effective ISB. We present a case of an unexpected anatomical variation of the ASM encountered while performing an ISB. Our patient had a small redundant ASM, which was challenging to identify and necessitated the use of a nerve stimulator to confirm the accurate needle placement and local anesthetic deposition.

## Case presentation

**Figure 1:**
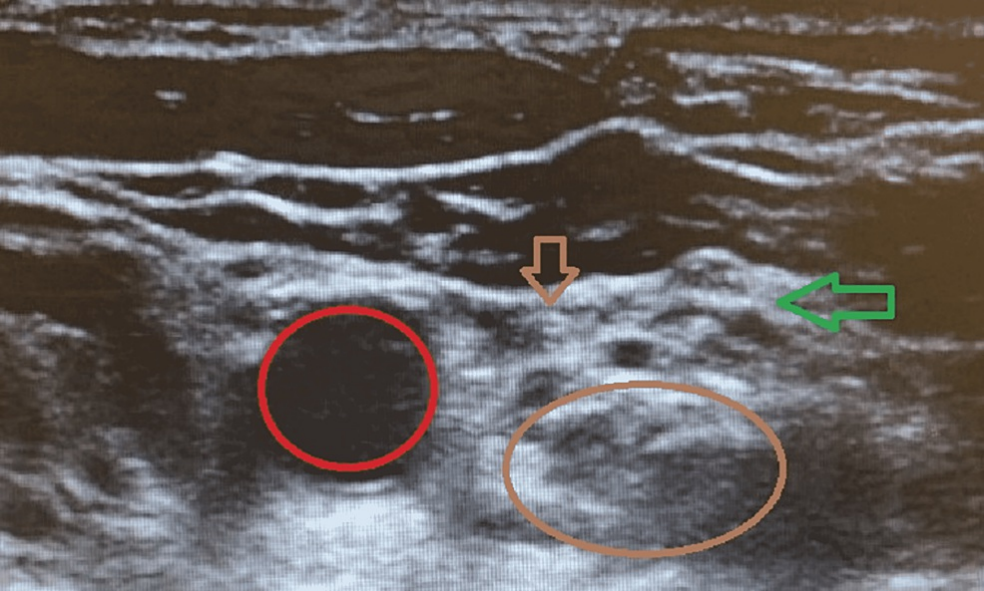
Ultrasound image of the brachial plexus nerve roots Red circle: carotid artery; brown circle: middle scalene muscle; green arrow: brachial plexus nerve roots; brown arrow: anterior scalene muscle

A 69-year-old female presented to our facility for an elective left total shoulder reverse arthroplasty. The patient complained of left shoulder pain that had started after suffering a fall. Shoulder X-ray and CT showed a closed fracture of the left humeral head. Our team formulated an anesthetic plan involving combined regional and general anesthesia. The patient then consented to an ultrasound-guided ISB. She was positioned supine in the regional block room, head directed towards the right, and angled slightly upwards. The ultrasound probe was applied in a transverse position at the level of C6 and medial to the sternomastoid muscle to identify the carotid artery. The ultrasound image showed unexpected anatomy, and multiple attempts at identifying the ASM in different views were unsuccessful. C5, C6, and seventh cervical (C7) brachial plexus nerve roots were visualized in the interscalene area, anterior to the middle scalene muscle, but the ASM was redundant and appeared smaller than anticipated (Figure 1).

A peripheral nerve stimulator was used to identify the nerve roots correctly, and a successful ISB was performed, with the patient subsequently reporting numbness in her left shoulder. She then underwent a successful left total reverse arthroplasty procedure and was admitted to the surgical ward postoperatively. The ISB adequately controlled her pain over the immediate postoperative period, allowing her to participate in physical therapy and helping her progress towards her goals effectively. She was then successfully discharged home.

## Discussion

This report discusses a case of a 69-year-old female who presented to our facility for an elective left shoulder reverse arthroplasty. An ultrasound-guided ISB was planned as part of the anesthetic management of the case. We followed standard techniques with ultrasound guidance to identify the brachial plexus in the interscalene groove. The ultrasound image showed an unexpected anatomical variation, with the ASM hardly recognizable, redundant, and smaller in size than anticipated. The ultrasound was then used to identify the brachial plexus in the supraclavicular area. Then the ultrasound probe was moved cranially, tracing the brachial plexus backward to the roots' level. We then confirmed that the small redundant muscular structure, previously seen with standard ultrasound technique and located medial to the brachial plexus roots, was the ASM. Before local anesthetic injection, a traditional nerve stimulator was used for further confirmation.

The brachial plexus is formed from the union of the ventral rami of spinal nerves C5 to T1. It passes through the interscalene groove along its course in the posterior triangle of the neck [8]. The interscalene groove is bounded anteriorly by the ASM, posteriorly by the MSM, and inferiorly by the first rib. The ISB is performed at the interscalene groove at the level of the sixth cervical vertebra, where the brachial plexus roots are found lateral to the internal jugular vein and carotid artery located in between the ASM and MSM.

There are two approaches employed for the ISB: the classic approach and the low ISB approach. In the classic approach, the patient is positioned supine. The head is turned away from the side of the block, and it is angled slightly upward. The index and middle fingers of the non-dominant hand are placed behind the lateral edge of the sternomastoid muscle and then moved medially till the ASM is felt. Palpation is then started in a lateral direction till the fingers are placed in the interscalene groove. A 22-gauge needle is then inserted between the index and middle fingers at the level of C6, perpendicular to the skin, and a local anesthetic is injected after negative aspiration [9]. In the low ISB approach, the interscalene groove is marked from the level of C6 to the clavicle, and the area is divided into three equal parts. The block is then performed using a 22-gauge needle at a distance two-thirds caudally from the level of C6 [10].

Brachial plexus anatomical variations are commonly encountered. Further understanding of the potential variations and their clinical implications is crucial for a safe and effective application of regional anesthesia. A small redundant ASM is a possible anatomical variation encountered on ultrasound imaging when an ISB is performed. We highlight an alternative approach to ultrasound-guided identification of the brachial plexus when such an anatomical variation is present. This approach can be made by identifying the brachial plexus in the supraclavicular area and tracing its course in a cranial direction to the nerve roots' level, followed by using a nerve stimulator for further confirmation of the needle placement.

## Conclusions

Anatomical variations of the brachial plexus are very common. Knowledge of possible anatomical variations is integral for a safe and effective practice of regional anesthesia. Although prior research has shown numerous possible variations of the brachial plexus, there is scarce data on anatomical variations involving the surrounding musculature. A small redundant ASM is a potential anatomical variation encountered while performing an ISB. We recommend identifying the brachial plexus in the supraclavicular area and then tracing its course in a cranial direction up to the roots' level, followed by confirmation of the needle placement by using a nerve stimulator before local anesthetic deposition.

## References

[1] Mian A, Chaudhry I, Huang R, Rizk E, Tubbs RS, Loukas M (2014). Brachial plexus anesthesia: a review of the relevant anatomy, complications, and anatomical variations. Clin Anat.

[2] Fayed M, Nowak K, Angappan S, Patel N, Abdulkarim F, Penning DH, Chhina AK (2022). Emergent surgical airway skills: time to re-evaluate the competencies. Cureus.

[3] Coşarcan SK, Gurkan Y, Doğan AT, Koyuncu Ö, Erçelen Ö (2021). Could interscalene block possibly be protective against cerebral ischemia during shoulder surgery in a beach chair position?. Cureus.

[4] Harry WG, Bennett JD, Guha SC (1997). Scalene muscles and the brachial plexus: anatomical variations and their clinical significance. Clin Anat.

[5] Natsis K, Totlis T, Tsikaras P, Anastasopoulos N, Skandalakis P, Koebke J (2006). Variations of the course of the upper trunk of the brachial plexus and their clinical significance for the thoracic outlet syndrome: a study on 93 cadavers. Am Surg.

[6] Feigl GC, Litz RJ, Marhofer P (2020). Anatomy of the brachial plexus and its implications for daily clinical practice: regional anesthesia is applied anatomy. Reg Anesth Pain Med.

[7] Lucas JP, Sandouka A, Rosenthal OD (2020). Coexistence of brachial plexus-anterior scalene and sciatic nerve-piriformis variants. Cureus.

[8] Aheer GK, Villella J (2021). Scalenus muscle and the C5 root of the brachial plexus: bilateral anatomical variation and its clinical significance. J Can Chiropr Assoc.

[9] Pester JM, Varacallo M (2022). Brachial Plexus Block Techniques. StatPearls. Published Online First: 12 February.

[10] Kim JH, Chen J, Bennett H (2011). A low approach to interscalene brachial plexus block results in more distal spread of sensory-motor coverage compared to the conventional approach. Anesth Analg.

